# Assessment of Primary Health Care Physicians’ Awareness, Knowledge, and Practice of Familial Hypercholesterolemia in Jazan, Saudi Arabia

**DOI:** 10.7759/cureus.87625

**Published:** 2025-07-09

**Authors:** Rahmah Bashiri, Mousa Mawkili

**Affiliations:** 1 Family Medicine, Ministry of Health Holdings, Jazan, SAU

**Keywords:** awareness, cardiovascular disease prevention, familial hypercholesterolemia, family physicians, knowledge, primary care, saudi arabia

## Abstract

Introduction

Familial hypercholesterolemia (FH) is a prevalent genetic disorder that predisposes affected individuals to premature coronary artery disease (CAD). Despite its clinical significance, global studies have revealed major deficits in awareness and knowledge among healthcare providers. This study aimed to assess the awareness, knowledge, and practices regarding FH among family physicians in primary health care centers in the Jazan region, Saudi Arabia.

Methods

A descriptive cross-sectional study was conducted among family physicians working at primary care centers in Jazan. Participants were recruited through convenience sampling using a validated, self-administered online questionnaire adapted from previous studies. The questionnaire addressed demographics, knowledge, awareness, and clinical practices related to FH. A p-value < 0.05 is considered statistically significant.

Results

The study included 123 participants, with a response rate of 62.1%. Most were male, 81 (66%), and 77 (64%) had ≤ 5 years of experience. A total of 102 (83%) correctly defined FH, while only 27 (22%) knew its prevalence. Knowledge of inheritance and cardiovascular risk was accurate in 37 (30%) and 15 (12%) participants, respectively. Cascade screening awareness was reported by 78 (63%), and 81 (66%) were aware of referral centers. Only 45 (36.6%) demonstrated acceptable knowledge. No significant associations were found between knowledge levels and demographic factors.

Conclusion

There is a substantial deficiency in FH-related knowledge among primary care physicians in Jazan. Targeted educational initiatives and continuous professional development are critically needed to enhance FH recognition and management in primary care settings.

## Introduction

Familial hypercholesterolemia (FH) is a common inherited lipid disorder characterized by markedly elevated levels of low-density lipoprotein cholesterol (LDL-C), which accelerates atherosclerotic plaque formation and significantly increases the risk of premature coronary artery disease (CAD) [1,2]. FH follows an autosomal dominant inheritance pattern and is most frequently associated with pathogenic variants in genes such as LDLR, APOB, and PCSK9 [2]. Clinically, FH presents in two forms: heterozygous (HeFH) and homozygous (HoFH), with HeFH accounting for an estimated 70-90% of cases [2,3].

Timely identification and management of FH critically depend on healthcare providers' awareness and knowledge. However, global data indicate that a large proportion of FH cases remain undiagnosed or inadequately treated, primarily due to limited professional awareness [4,5]. This knowledge gap compromises early diagnosis, delays the initiation of therapy, and reduces the effectiveness of cascade screening for affected family members [6,7]. Even when physicians are generally familiar with FH, adherence to clinical guidelines for diagnosis and management is often suboptimal, resulting in inconsistent care and substandard outcomes [5,8,9].

A study among Malaysian primary care physicians (PCPs) demonstrated significantly higher knowledge (53.5 ± 13.9 vs. 35.9 ± 11.79, p < 0.001) and practice (69.2 ± 17.62 vs. 54.4 ± 19.28, p < 0.001) scores among those holding postgraduate qualifications. Similarly, awareness scores were higher in this group (15.4 vs. 7.7, p = 0.030) [7]. Comparable deficits in FH-related knowledge and practice have been documented among physicians in Riyadh, Saudi Arabia [10]; emergency care providers in Melbourne, Australia [11]; and registered practitioners in India [12].

In Riyadh, only 51.6% of family physicians demonstrated an acceptable level of FH knowledge [13]. Likewise, a survey of Saudi medical interns revealed that while understanding of the FH definition (76.5%) and basic lipid profile (52.4%) was relatively adequate, knowledge of inheritance patterns (43.5%), disease prevalence (12.4%), and CAD risk (7.1%) was insufficient [14].

Professional training and continuous medical education have been widely endorsed as key strategies for bridging these gaps, enhancing early detection, and ensuring evidence-based care [5,8]. Improved provider knowledge directly correlates with better patient outcomes, more effective family screening, and a reduced cardiovascular disease burden on public health systems [15,16]. Accordingly, the present study aims to evaluate the levels of awareness, knowledge, and clinical practice regarding FH among primary healthcare physicians in the Jazan region of Saudi Arabia.

## Materials and methods

Study design and area 

This observational descriptive cross-sectional study was conducted at Primary Healthcare Centers, Jazan Health Cluster, located in the southwestern part of Saudi Arabia along the Red Sea coast. According to the General Authority for Statistics, the region's population was estimated at approximately 1.4 million as of 2022. In Jazan, 552 physicians are employed across 165 primary healthcare centers, distributed among eight sectors under the Ministry of Health.

Study population 

The study included all family physicians under 60 years of age working in primary healthcare centers affiliated with the Ministry of Health in Jazan. Physicians from other specialties, healthcare professionals other than family physicians, and those working in health facilities under the armed or security forces were excluded.

Sample size 

The sample size was calculated based on a previous study conducted in Riyadh, where 72.4% of participants reported an average or above-average familiarity with FH [13]. Using a 5% margin of error, a 95% confidence interval, a proportion of 72.4%, and a population of 552, the required sample size was 198. Accounting for a 20% non-response rate, the final sample size was adjusted to 238 participants.

Sampling technique and instrument

Data was collected conveniently between December 2024 and April 2025. A self-administered online questionnaire was distributed via Google Forms (Google LLC, Mountain View, California, United States) to sector coordinators, who forwarded it to family physicians in their respective sectors (Appendix 1). To ensure face validity, a panel of experts reviewed the questionnaire. It was adapted from validated instruments used in previous Saudi studies [10,13,14,17], originally developed by Bell et al. [18], with additional questions from Batais et al. [10], based on expert recommendations and international FH guidelines. Approval to use the questionnaire in this study was obtained from Dr. Bell and Dr. Batais. Both provided written permission to adapt and utilize the instrument for research purposes. The questionnaire comprised two sections. The first covered demographics, including gender, qualifications, training status, and years of practice. The second focused on FH-related knowledge, awareness, detection, and clinical practices. All questions were multiple-choice; no open-ended questions were included.

Statistical analysis plan

Data were collected in Excel, cleaned, and analyzed using the IBM SPSS Statistics for Windows, Version 26 (Released 2018; IBM Corp., Armonk, New York, United States). Descriptive statistics were used to assess FH knowledge. Categorical variables were reported as frequencies and percentages, while continuous variables were presented using the median and interquartile range. Each correct answer was coded as one, and each incorrect answer as zero. Knowledge scores were calculated by summing all correct responses. Participants who scored ≥ 50% of correct answers were considered to have an acceptable knowledge level. The internal consistency of the questionnaire was assessed using Cronbach’s alpha, which was found to be 0.603. Fisher’s exact test and Pearson’s chi-squared test were used to determine associations between knowledge and demographic factors. A p-value < 0.05 was considered statistically significant.

Ethical consideration

This study was conducted following Saudi ethical standards and was reviewed by the Research Ethics Committee of Jazan, approval number: 2499. Informed consent was obtained via a checkbox in the Google Form. Participant confidentiality and anonymity were strictly maintained.

## Results

A total of 123 healthcare professionals participated, yielding a response rate of 62.1%. Fifty-four (44%) were aged ≤ 30 years, and 60 (49%) were aged 31-45 years. The majority were male (81; 66%), and most were Saudi nationals (98; 80%). Regarding professional rank, 51 (41%) were residents, 30 (24%) were general practitioners, and 22 (18%) were senior registrars. Geographically, most respondents were from the southern (28; 23%) and western (24; 20%) regions. Sixty-four percent (77) had ≤ 5 years of experience, while 24 (20%) had six to 10 years of experience (Table 1).

**Table 1:** Demographic characteristics of healthcare professionals ^1 ^Data were presented as median (IQR) or n (%)

Characteristic	N = 123^1^
Age	
≤30	54 (44%)
31-45	60 (49%)
>45	9 (7.3%)
Gender	
Female	42 (34%)
Male	81 (66%)
Nationality	
Non-Saudi	25 (20%)
Saudi	98 (80%)
Level of Training	
General practitioners	30 (24%)
Resident	51 (41%)
Registrar	7 (5.7%)
Senior registrar	22 (18%)
Consultant	13 (11%)
Health Sector	
Central	21 (17%)
Middle	22 (18%)
Northern	13 (11%)
Southern	28 (23%)
Western	24 (20%)
Jabali	4 (3.3%)
Bani Malik	11 (8.9%)
Experience Year	
≤5	77 (64%)
6-10	24 (20%)
11-14	12 (9.9%)
≥16	8 (6.6%)
Unknown	2

Of the 123 participants, 102 (83%) correctly identified the definition of FH. Only 27 (22%) knew that the prevalence of HeFH in the general population is one in 500. Thirty-seven (30%) correctly recognized that first-degree relatives of FH patients have a 50% chance of inheriting the condition. Only 15 (12%) acknowledged that untreated FH patients have a 20-fold higher risk of premature coronary heart disease. Regarding the age thresholds for premature cardiovascular disease, 37 (33%) correctly identified 55 years for males, and 29 (25%) identified 65 years for females. Additionally, 64 (52%) knew that the FH diagnosis is not solely based on genetic testing.

As for treatment targets, 59 (48%) identified an LDL-C goal of < 2.5 mmol/L for adults with FH, and 89 (72%) recognized a target of < 1.8 mmol/L for those with CAD or diabetes. A majority (93; 76%) knew that statins are the first-line treatment, and 69 (56%) correctly indicated the use of statins with ezetimibe for severe hypercholesterolemia (Table 2).

**Table 2:** Knowledge of familial hypercholesterolemia among healthcare professionals ^1^Data were presented as n (%) FH: familial hypercholesterolemia; CVD: cardiovascular disease; LDL: low-density lipoprotein cholesterol; CHD: congenital heart disease

Characteristic	N = 123^1^
Correct definition of FH	102 (83%)
Every 1 in 500 person is the prevalence of heterozygous FH in the general population	27 (22%)
There is a 50% likelihood that first-degree relatives of someone who has FH will also have FH themselves	37 (30%)
There is a 20 times greater risk of premature coronary heart disease in untreated FH patients compared to the general population	15 (12%)
55 years old is the age considered premature for CVD in male patients with a family history of FH	37 (33%)
65 years old is the age considered premature for CVD in female patients with a family history of FH	29 (25%)
An accurate diagnosis of FH can only be made via genetic testing; the statement is false	64 (52%)
An LDL target of < 2.5 mmol/L for adults with FH	59 (48%)
An LDL target of < 1.8 mmol/L for FH adults with known CHD or diabetes	89 (72%)
Patients with FH should receive statins as first-line treatment	93 (76%)
Statins and ezetimibe combinations are used to treat severe hypercholesterolemia	69 (56%)

Regarding cascade screening, 78 (63%) were aware of its use in FH. Concerning diagnostic criteria, 50 (41%) were familiar with the Dutch Lipid Clinic Network criteria, 50 (41%) with the US MedPed Program, and 23 (19%) with the Simon Broome criteria. However, 53 (43%) were unaware of any of these diagnostic tools. When asked about non-statin treatments, 19 (15%) cited mipomersen, 71 (58%) noted PCSK9 inhibitors, and 35 (28%) mentioned lomitapide. Still, 35 (28%) were unaware of any alternatives. Eighty-one (66%) knew of referral centers for lipid disorders.

When asked about routine assessments in premature CAD patients, 98 (80%) selected all options. However, only seven (5.7%) reported examining for arcus cornealis, and another seven (5.7%) for tendon xanthomata. Few reported screening close relatives (4; 3.3%) or taking a detailed family history (5; 4.1%). Regarding cardiovascular risk factors in FH, 63 (51%) selected lipoprotein (A), 87 (71%) smoking, 27 (22%) C-reactive protein, and 84 (68%) type 2 diabetes. Only three (2.4%) selected “none of the above,” while nine (7.3%) responded “don’t know.”

As for tools aiding FH detection, 38 (31%) mentioned lab comments, 20 (16%) clinical software alerts, and eight (6.5%) telephone alerts from labs. Seventy-seven (63%) selected all of the above; two (1.6%) selected none; and 10 (8.1%) responded “don’t know” (Table 3).

**Table 3:** Awareness of familial hypercholesterolaemia ^1^Data were presented as n (%) FH: familial hypercholesterolemia; CAD: coronary artery disease

Characteristic	N = 123^1^
Aware of the cascade screening for patients with FH	78 (63%)
Aware of the clinical diagnostic algorithm to diagnose patients with FH	
The Dutch Lipid Clinic Network criteria	50 (41%)
The US MedPed Program	50 (41%)
The Simon Broome criteria	23 (19%)
None of them	53 (43%)
Aware of medication to treat FH besides statins	
Mipomersen (an antisense oligonucleotide inhibitor)	19 (15%)
PCSK9 inhibitors	71 (58%)
Lomitapide (MTP inhibitor)	35 (28%)
None of them	35 (28%)
Aware of any specialist clinical services for lipid disorders to whom you can refer patients	81 (66%)
Routinely carry out in patients with documented premature coronary artery disease	
All of the above	98 (80%)
Examine for arcus cornealis	7 (5.7%)
Examine for tendon xanthomata	7 (5.7%)
None of the above	2 (1.6%)
Screen close relatives for hypercholesterolemia	4 (3.3%)
Take a detailed family history of coronary artery disease	5 (4.1%)
Factors that increase the risk of cardiovascular disease in patients with FH	
Elevated lipoprotein A	63 (51%)
Smoking	87 (71%)
Elevated C-reactive protein	27 (22%)
Type 2 diabetes mellitus	84 (68%)
None of the above	3 (2.4%)
Do not know	9 (7.3%)
Factors that help in the detection of FH in your practice	
Laboratory comment on lipid profile alerting to a possible FH	38 (31%)
Alert by the clinical software system in your practice	20 (16%)
Direct telephone call from the laboratory	8 (6.5%)
All of the above	77 (63%)
None of the above	2 (1.6%)
Do not know	10 (8.1%)

Thirty-six (29%) had diagnosed FH, and 38 (31%) had followed up on FH cases. Most (68; 55%) routinely screened patients’ children and close relatives, while nine (7.3%) screened children only. Forty-one (33%) tested at-risk youth aged 13-18 years, and 32 (26%) tested those aged seven to 12 years (Table 4).

**Table 4:** Practice related to familial hypercholesterolemia among healthcare professionals ^1^Data were presented as n (%) FH: familial hypercholesterolemia

Characteristic	N = 123^1^
Ever diagnosed a patient with FH	36 (29%)
Ever followed a patient with FH	38 (31%)
Routinely screen close relatives of patients with FH for FH with lipid profile	
No	21 (17%)
Not applicable	25 (20%)
Yes, the patient’s children and other close relatives	68 (55%)
Yes, the patient’s children only	9 (7.3%)
Age to test a young individual for hypercholesterolemia in a family with premature coronary heart disease	
0-6 years	13 (11%)
7-12 years	32 (26%)
13-18 years	41 (33%)
Don’t know	27 (22%)
None of the above	10 (8.1%)

**Figure 1:**
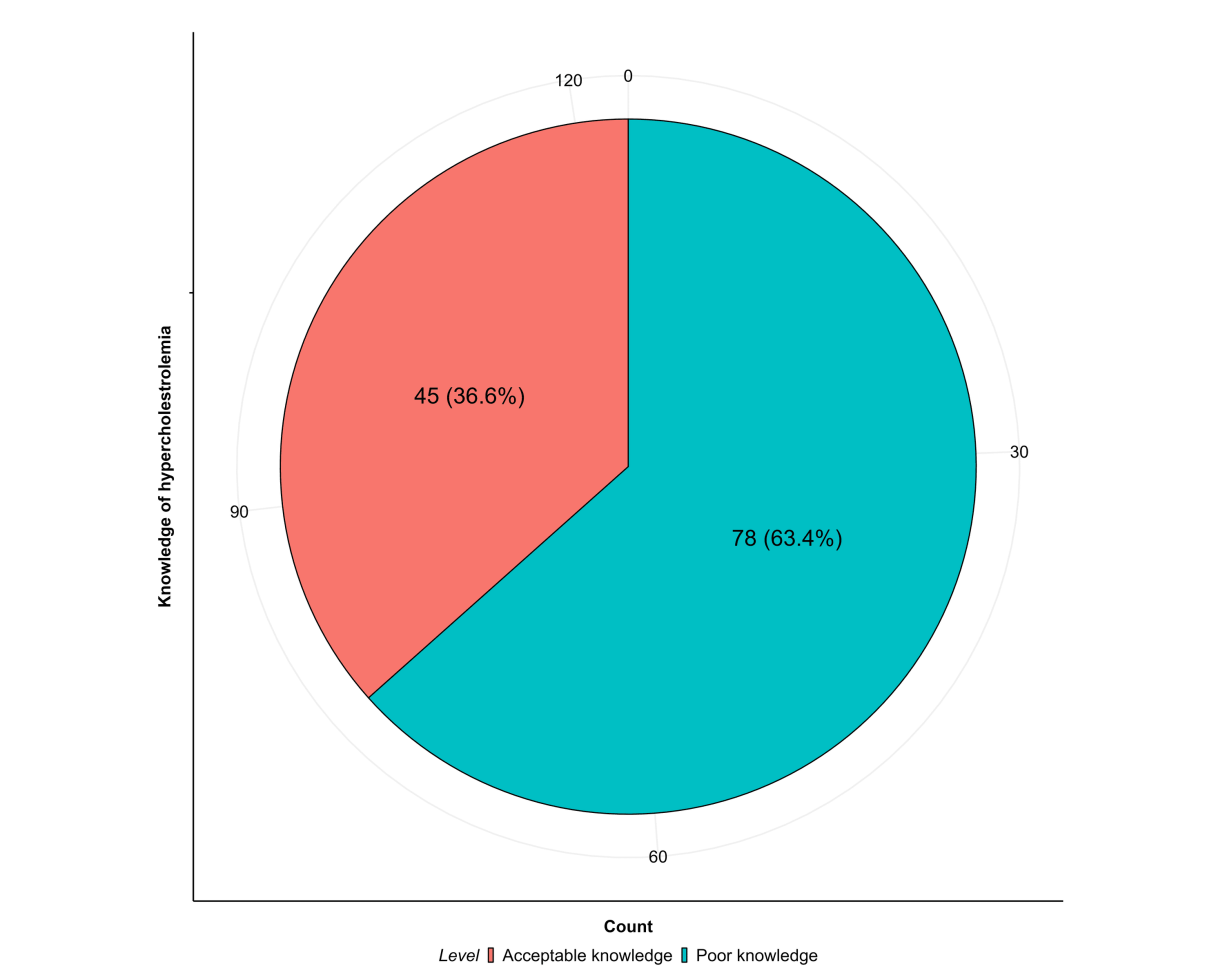
Knowledge level of familial hypercholesterolemia among healthcare professionals

The median knowledge score was five (4,6) and the mean score was 5.11 ± 2.3. Only 45 (36.6%) participants had an acceptable knowledge level, while 78 (63.4%) scored poorly (Figure 1).

Knowledge was highest among participants aged > 45 years (44.4% vs. 31.6% vs. 40.7%; p = 0.5), males (39.5% vs. 30.9%; p = 0.4), and Saudis (38.8% vs. 28%). Consultants had the highest acceptable knowledge rate (53.8%; p = 0.5). Participants from the western region scored better (p = 0.14), and those with six to 10 years of experience outperformed others (p = 0.8). However, no demographic factor showed a statistically significant association with knowledge (Table 5).

**Table 5:** Association between knowledge of familial hypercholesterolemia and demographic characteristics ^1^Data were presented as n (%) *: Fisher's exact test; #: Pearson's chi-squared test

Characteristic	Acceptable knowledge, N = 45^1^	Poor knowledge, N = 78^1^	Statistic	p-value
Age*			1.267	0.5
≤30	22 (49%)	32 (41%)		
31-45	19 (42%)	41 (53%)		
>45	4 (8.9%)	5 (6.4%)		
Gender#			0.542	0.4
Female	13 (29%)	29 (37%)		
Male	32 (71%)	49 (63%)		
Nationality#			0.587	0.3
Non-Saudi	7 (16%)	18 (23%)		
Saudi	38 (84%)	60 (77%)		
Level of Training*			3.428	0.5
Consultant	7 (16%)	6 (7.7%)		
General practitioners	8 (18%)	22 (28%)		
Registrar	3 (6.7%)	4 (5.1%)		
Resident	20 (44%)	31 (40%)		
Senior registrar	7 (16%)	15 (19%)		
Health Sector*			9.568	0.14
Bani Malik	1 (2.2%)	10 (13%)		
Central	6 (13%)	15 (19%)		
Jabali	2 (4.4%)	2 (2.6%)		
Middle	8 (18%)	14 (18%)		
Northern	4 (8.9%)	9 (12%)		
Southern	10 (22%)	18 (23%)		
Western	14 (31%)	10 (13%)		
Experience Year*			0.993	0.8
≤5	27 (60%)	50 (66%)		
6-10	11 (24%)	13 (17%)		
11-14	4 (8.9%)	8 (11%)		
≥16	3 (6.7%)	5 (6.6%)		

## Discussion

FH is a genetic disorder characterized by elevated levels of LDL-C in the blood [19]. A study reported that the estimated FH prevalence in the Arabian Gulf region was 0.9% (1:112) [20]. Although no epidemiological studies have yet documented the exact prevalence of FH in Saudi Arabia, estimates suggest that between 63,485 and 158,712 individuals may be affected [21]. The high rate of consanguinity and underreporting of FH mutations in Saudi Arabia [13] further complicate identification and management, placing considerable responsibility on physicians to recognize and address the condition effectively. Accordingly, this study was conducted to evaluate knowledge, awareness, and clinical practices related to FH among primary healthcare physicians in Jazan, Saudi Arabia.

Our study revealed that nearly two-thirds of participants had poor knowledge about FH, along with notable deficiencies in awareness and significant gaps in clinical practice. These findings are concerning, given the critical role primary healthcare physicians play in early detection and management.

The overall knowledge level observed in this study was slightly higher than that reported in a previous study from Jeddah [14], but lower than in a Riyadh-based study [13], indicating regional variations. These discrepancies may reflect differences in training, access to continuing medical education, and exposure to lipid disorders. Despite being conducted in different parts of the same country, the findings highlight the need for a unified, standardized approach to FH education and management nationwide. Further studies are required to identify the root causes of these disparities and to develop targeted interventions for both healthcare providers and the general population.

While participants demonstrated limited knowledge regarding FH prevalence, inheritance, and the age thresholds for premature cardiovascular disease onset, these findings were more favorable than those reported in an Australian study [18]. Encouragingly, over 80% of participants correctly defined FH, slightly below the rate among Malaysian PCPs [7], and approximately three-quarters identified statins as first-line therapy, which is higher than in some earlier Saudi studies [10]. These results suggest that while treatment-related knowledge exists, awareness regarding detection and screening remains inadequate.

No statistically significant associations were found between knowledge levels and demographic variables. Nonetheless, older consultants demonstrated higher knowledge levels, suggesting that clinical experience may enhance familiarity with FH.

Regarding awareness, the study found major gaps, particularly in familiarity with diagnostic criteria and alternative treatment options such as mipomersen. These findings align with previous research from Riyadh, the UK, and the Asia-Pacific region [4,13,22]. Awareness of diagnostic tools and emerging therapies is essential for effective FH management, and efforts to improve this knowledge are urgently needed.

Positively, many physicians demonstrated good awareness of routine assessments for patients with premature CAD. Nearly two-thirds were familiar with cascade screening and aware of referral options to specialized lipid clinics, levels notably higher than in an Indian study [12]. Participants also accurately identified cardiovascular risk factors such as smoking and type 2 diabetes, consistent with prior Saudi research [13].

Approximately one-third of participants reported diagnosing or managing FH cases, suggesting that FH remains under-recognized in primary care. This is consistent with findings from the Riyadh study [13] and points to missed opportunities for early intervention. Encouragingly, over half of respondents reported screening children and first-degree relatives, in line with clinical guidelines recommending lipid testing (with or without genetic testing) for all first-degree relatives [23].

As the first study of its kind in the Jazan region, this research provides valuable baseline data on FH-related knowledge, awareness, and practices among PCPs. This focus is particularly important, as these professionals serve as the initial point of contact for most patients and play a key role in the early detection and management of chronic conditions such as FH.

However, this study has certain limitations. The cross-sectional design and reliance on convenience sampling limit the generalizability of our findings to the broader population of primary healthcare physicians in Saudi Arabia. Additionally, we did not conduct a non-responder bias analysis, which could have provided insights into whether the views of respondents systematically differed from those who chose not to participate. The use of self-reported data may also introduce response bias, as perceived knowledge may not accurately reflect actual knowledge. Finally, the study’s regional scope restricts the applicability of its findings to other parts of Saudi Arabia.

## Conclusions

FH represents a significant public health concern in Saudi Arabia, particularly given its genetic basis and the country’s high rate of consanguinity. This study found that nearly two-thirds of primary healthcare physicians in the Jazan region demonstrated poor knowledge of FH, along with considerable deficiencies in awareness and clinical practice. These findings underscore the urgent need for targeted and sustained educational programs for primary care providers, who are crucial to early detection and long-term management.

Incorporating FH-focused content into continuing medical education and developing national guidelines tailored to primary care settings may improve knowledge and reduce regional disparities. Additionally, promoting clinical exposure through structured rotations, case-based learning, and referral systems may enhance early identification and optimize patient outcomes.

## Appendices

Appendix 1

Questionnaire

Section 1: Physician’s Demographics

  1. What is your age?                                                                                                                                                   

Years ــــ

2. What is your gender?

o Male

o Female

3. What is your level of training?

o Resident

o Registrar

o Consultant

o General practice

o Senior registrar

4. How many years have you been in practice since completing your medical school?

_______# of years

Section 2:

5. How familiar are you with familial hypercholesterolemia? Please use a 10-point scale where 1 means “Not at all familiar” and 10 means “Extremely familiar”.

1 2 3 4 5 6 7 8 9 10

The following questions are specific to familial hypercholesterolemia. Please respond to the best of your knowledge, and if you are unsure of any question, select “Don’t know.”

6. Which one descriptions below best describes familial hypercholesterolemia?

o The presence of family members with diagnosed high cholesterol

o A genetic disorder that is characterized by very high cholesterol and a family history of premature heart disease

o The presence of multiple lipid abnormalities that may be genetic in nature

o An ultra-rare, potentially fatal condition caused by cholesterol levels that can be up to six times the normal level

o Don’t know

7. What is the prevalence of heterozygous familial hypercholesterolemia in the general population?

o 1 in 100 persons

o 1 in 500 persons

o 1 in 1,000 persons

o 1 in 2,000 persons

o 1 in 5,000 persons

o Don’t know

8. What is the likelihood that first-degree relatives (i.e., parents, siblings, and children) of someone who has familial hypercholesterolemia will also have FH themselves?

o 0%

o 25%

o 50%

o 75%

o 100%

o Don’t know

9. Across all age groups, how much greater is the risk of premature coronary heart disease (CHD) in untreated familial hypercholesterolemia patients compared to the general population? Please answer to the best of your knowledge.

o 2 times greater

o 5 times greater

o 10 times greater

o 20 times greater

o Don’t know25

10. When you are assessing a patient’s family history, at what age for males and females do you consider heart disease to be premature?

o Premature heart disease in Males: ____# Years of age or younger

o Premature heart disease in Females: ____# Years of age or younger

o Don’t know

11. In patients with documented premature coronary artery disease, which one of the following do you routinely carry out?

o Examine for arcus cornealis

o Examine for tendon xanthomata

o Take a detailed family history of coronary artery disease

o Screen close relatives for hypercholesterolemia

o All of the above

o None of the above

12. An accurate diagnosis of FH can only be made via genetic testing. Is this statement true or false?

o True

o False

o Don’t know

13. Which, if any, of the following have been recognized to further increase the cardiovascular risk of someone with familial hypercholesterolemia? Please select all that apply.

o Elevated lipoprotein(a)

o Smoking

o Elevated C-reactive protein

o Type 2 diabetes

o None of the above

o Don’t know

14. In your view, which healthcare providers would be most effective at the early detection of familial hypercholesterolemia and screening first-degree relatives? Please select up to two.

o Lipid specialists

o Primary care physicians

o Cardiologists

o Pediatricians

o Obstetrics and gynecology

o Endocrinologists

o Family medicine

15. Have you ever diagnosed a patient with FH?

o Yes

o No

16. Have you ever followed a patient with FH?

o Yes

o No

o Don’t remember

17. Which one of the following choices could help you in the detection of FH in your practice?

o Laboratory comment on lipid profile alerting to a possible FH

o Alert by the clinical software system in your practice

o Direct telephone call from the laboratory

o All of the above

o None of the Above

o Other (please specify)

o Don’t know

18. If you have patients with FH under your care, do you routinely screen their close relatives for this condition with a lipid profile?

o Yes, the patient’s children only

o Yes, the patient’s children and other close relatives

o No

o Not applicable

19. Are you aware of these Clinical diagnostic algorithms to diagnose patients with FH?

o The Simon Broome criteria

o The Dutch Lipid Clinic

o Network DLCN criteria

o The US MedPed Program

o none of them

20. At what age would you test a young individual for hypercholesterolemia in a family with premature coronary heart disease?

o 0-6 years

o 7-12 years

o 13-18 years

o None of the above

o Don’t know

21. Are you aware of the cascade screening for patients with familial hypercholesterolemia?

o Yes

o No

22. Are you aware of any specialist clinical services for lipid disorders to which you can refer patients?

o Yes

o No

23. An LDL target for adults with FH is:

o < 1.8 mmol/L (< 70 mg/dL)

o < 2.5 mmol/L (< 100 mg/dL)

o < 3.3 mmol/L (< 130 mg/dL)

o Don’t know28

24. An LDL target for FH adults with known CHD or diabetes is:

o < 1.8 mmol/L (< 70 mg/dL)

o < 2.5 mmol/L (< 100 mg/dL)

o < 3.3 mmol/L (< 130 mg/dL)

o Don’t know

25. Patients with FH should receive which one of the following medications as first-line treatment? Please select one option only.

o Exchange resins/bile acid sequestrates

o Ezetimibe

o Statins

o Fibrates

o Nicotinic acid

o Don’t know

26. Which drug combinations do you use to treat severe hypercholesterolemia? Please select all that apply

o Statin + Exchange resins/bile acid sequestrates

o Statin + Nicotinic acid

o Statin + Ezetimibe

o Statin + Ezetimibe+ Nicotinic acid

o Statin + Ezetimibe + Exchange resins/bile acid sequestrates

o Other (please specify)

o None of the above

27. Are you aware of these medications for FH patients besides statins?

o PCSK9 inhibitors 

o Lomitapide (MTP) inhibitor. 

o Mipomersen (an antisense ligonucleotide inhibitor)

o none of the above

* Approval to use the questionnaire in this study was obtained from Dr. Bell and Dr. Batais. Both provided written permission to adapt and utilize the instrument for research purposes.

## References

[1] Najam O, Ray KK (2015). Assessment of primary health care physicians’ awareness, knowledge, and practice of familial hypercholesterolemia in Jazan, Saudi Arabia. Cardiol Ther.

[2] Austin MA, Hutter CM, Zimmern RL, Humphries SE (2004). Genetic causes of monogenic heterozygous familial hypercholesterolemia: a HuGE prevalence review. Am J Epidemiol.

[3] De Castro-Orós I, Pocoví M, Civeira F (2010). The genetic basis of familial hypercholesterolemia: inheritance, linkage, and mutations. Appl Clin Genet.

[4] Pang J, Sullivan DR, Harada-Shiba M, Ding PY, Selvey S, Ali S, Watts GF (2015). Significant gaps in awareness of familial hypercholesterolemia among physicians in selected Asia-Pacific countries: a pilot study. J Clin Lipidol.

[5] Wilemon KA, Patel J, Aguilar-Salinas C (2020). Reducing the clinical and public health burden of familial hypercholesterolemia: a global call to action. JAMA Cardiol.

[6] Kalia I, Liang L, Shope R, Reilly M, Schwartz L (2024). Knowledge of familial hypercholesterolemia among cardiology healthcare providers. J Clin Transl Sci.

[7] Azraii AB, Ramli AS, Ismail Z (2018). Knowledge, awareness and practice regarding familial hypercholesterolaemia among primary care physicians in Malaysia: The importance of professional training. Atherosclerosis.

[8] Khera AV, Hegele RA (2020). What is familial hypercholesterolemia, and why does it matter?. Circulation.

[9] Kindt I, Mata P, Knowles JW (2017). The role of registries and genetic databases in familial hypercholesterolemia. Curr Opin Lipidol.

[10] Batais MA, Almigbal TH, Bin Abdulhak AA, Altaradi HB, AlHabib KF (2017). Assessment of physicians' awareness and knowledge of familial hypercholesterolemia in Saudi Arabia: is there a gap?. PLoS One.

[11] Mirzaee S, Rashid HN, Tumur O (2019). Awareness of familial hypercholesterolemia among healthcare providers involved in the management of acute coronary syndrome in Victoria, Australia. CJC Open.

[12] Rangarajan N, Balasubramanian S, Pang J, Watts GF (2016). Knowledge and awareness of familial hypercholesterolaemia among registered medical practitioners in Tamil Nadu: are they suboptimal?. J Clin Diagn Res.

[13] Arnous MM, Alghamdi AM, Ghoraba MA (2019). Assessment of family physicians' awareness and knowledge of familial hypercholesterolemia in governmental hospitals in Riyadh, Saudi Arabia. J Family Med Prim Care.

[14] Alzahrani SH, Bima A, Algethami MR, Awan Z (2020). Assessment of medical intern's knowledge, awareness and practice of familial hypercholesterolemia at academic institutes in Jeddah, Saudi Arabia. Lipids Health Dis.

[15] Watts GF, Gidding SS, Mata P (2020). Familial hypercholesterolaemia: evolving knowledge for designing adaptive models of care. Nat Rev Cardiol.

[16] Goldberg AC, Hopkins PN, Toth PP (2011). Familial hypercholesterolemia: screening, diagnosis and management of pediatric and adult patients: clinical guidance from the National Lipid Association Expert Panel on Familial Hypercholesterolemia. J Clin Lipidol.

[17] Alaqeel A, Alrashidi A (2022). Gaps in knowledge and practice for familial hypercholesterolemia among physicians caring for children in Saudi Arabia. Eur Rev Med Pharmacol Sci.

[18] Bell DA, Garton-Smith J, Vickery A, Kirke AB, Pang J, Bates TR, Watts GF (2014). Familial hypercholesterolaemia in primary care: knowledge and practices among general practitioners in Western Australia. Heart Lung Circ.

[19] (2025). About familial hypercholesterolemia. https://www.cdc.gov/heart-disease-family-history/about/about-familial-hypercholesterolemia.html.

[20] Alhabib KF, Al-Rasadi K, Almigbal TH (2021). Familial hypercholesterolemia in the Arabian Gulf region: clinical results of the Gulf FH Registry. PLoS One.

[21] DIAGNOSIS AND TREATMENT OF FAMILIAL HYPERCHOLESTEROLEMIA IN SAUDI ARABIA (2025). Diagnosis and Treatment of Familial Hypercholesterolemia in Saudi Arabia: Clinical Protocol. https://www.moh.gov.sa/Ministry/MediaCenter/Publications/Documents/Protocol-019.pdf.

[22] Schofield J, Kwok S, France M (2016). Knowledge gaps in the management of familial hypercholesterolaemia. A UK based survey. Atherosclerosis.

[23] Brumit ML (2024). Diagnostic criteria for familial hypercholesterolemia. Family Heart Foundation.

